# A P300 Brain-Computer Interface With a Reduced Visual Field

**DOI:** 10.3389/fnins.2020.604629

**Published:** 2020-12-03

**Authors:** Luiza Kirasirova, Vladimir Bulanov, Alexei Ossadtchi, Alexander Kolsanov, Vasily Pyatin, Mikhail Lebedev

**Affiliations:** ^1^Samara State Medical University, Samara, Russia; ^2^Laboratory of Mathematical Processing of Biological Information, IT Universe Ltd, Samara, Russia; ^3^Center for Bioelectric Interfaces of the Institute for Cognitive Neuroscience, National Research University Higher School of Economics, Moscow, Russia; ^4^Department of Information and Internet Technologies of Digital Health Institute, I.M. Sechenov First Moscow State Medical University, Moscow, Russia; ^5^Center For Neurobiology and Brain Restoration, Skolkovo Institute of Science and Technology, Moscow, Russia

**Keywords:** P300 BCI, aperture, central vision, ERP, visual fatigue, visual attention

## Abstract

A P300 brain-computer interface (BCI) is a paradigm, where text characters are decoded from event-related potentials (ERPs). In a popular implementation, called P300 speller, a subject looks at a display where characters are flashing and selects one character by attending to it. The selection is recognized as the item with the strongest ERP. The speller performs well when cortical responses to target and non-target stimuli are sufficiently different. Although many strategies have been proposed for improving the BCI spelling, a relatively simple one received insufficient attention in the literature: reduction of the visual field to diminish the contribution from non-target stimuli. Previously, this idea was implemented in a single-stimulus switch that issued an urgent command like stopping a robot. To tackle this approach further, we ran a pilot experiment where ten subjects operated a traditional P300 speller or wore a binocular aperture that confined their sight to the central visual field. As intended, visual field restriction resulted in a replacement of non-target ERPs with EEG rhythms asynchronous to stimulus periodicity. Changes in target ERPs were found in half of the subjects and were individually variable. While classification accuracy was slightly better for the aperture condition (84.3 ± 2.9%, mean ± standard error) than the no-aperture condition (81.0 ± 2.6%), this difference was not statistically significant for the entire sample of subjects (*N* = 10). For both the aperture and no-aperture conditions, classification accuracy improved over 4 days of training, more so for the aperture condition (from 72.0 ± 6.3% to 87.0 ± 3.9% and from 72.0 ± 5.6% to 97.0 ± 2.2% for the no-aperture and aperture conditions, respectively). Although in this study BCI performance was not substantially altered, we suggest that with further refinement this approach could speed up BCI operations and reduce user fatigue. Additionally, instead of wearing an aperture, non-targets could be removed algorithmically or with a hybrid interface that utilizes an eye tracker. We further discuss how a P300 speller could be improved by taking advantage of the different physiological properties of the central and peripheral vision. Finally, we suggest that the proposed experimental approach could be used in basic research on the mechanisms of visual processing.

## Introduction

Following the introduction of P300 speller in 1988 by Farwell and Donchin (1988), many studies have strived to improve this method (Fazel-Rezai et al., 2012; Allison et al., 2020; Philip and George, 2020). The speller performance is hindered by the necessity to run many trials to distinguish target and non-target stimuli based on the comparison of the event-related potentials (ERPs) they evoke. Particularly, non-target items that are adjacent to the target attract attention and interfere with the decoding performance (Fazel-Rezai, 2007; Townsend et al., 2010). Several solutions to this problem have been explored, including using flashes of single items instead of flashing rows and columns (Guger et al., 2009), rearranging the spatial configuration of the simultaneously flashing stimuli (Townsend et al., 2010), suppressing the stimuli adjacent to targets (Frye et al., 2011), or all non-targets (Shishkin et al., 2011) during the calibration procedure, and optimizing the characteristics of visual stimuli (Salvaris and Sepulveda, 2009; Jin et al., 2017; Mainsah et al., 2017; Philip and George, 2020). Yet, all these approaches require a considerable amount of distracting stimuli for accurate spelling, which slows the decoding, and causes user fatigue (Boksem et al., 2005; Käthner et al., 2014; Oken et al., 2018).

In this perspective, we discuss a rather straightforward way to reduce the interference from non-target stimuli by the restriction of user sight to only the central visual field. Since the contribution from non-targets is blocked, the response to target stimulus could become cleaner and easier to detect. Previously, somewhat similar ideas were implemented in single-target brain-computer interfaces (BCIs) for generating an urgent command like braking a neurally-controlled wheelchair (Rebsamen et al., 2010) or stopping a robot (Fedorova et al., 2014). In these implementations, the speed of operation increased because the decoding was reduced to detecting the presence or absence of a single target stimulus. Here we tackled a different approach, where non-targets were effectively removed while the BCI was used for spelling instead of issuing a single command. We have conducted a pilot experiment where visual field reduction was accomplished by wearing a binocular aperture. When looking at the screen through the aperture, subjects were able to perform the same spelling task as they executed with the traditional P300-speller.

## Speller Settings With and Without the Aperture

**FIGURE 1:**
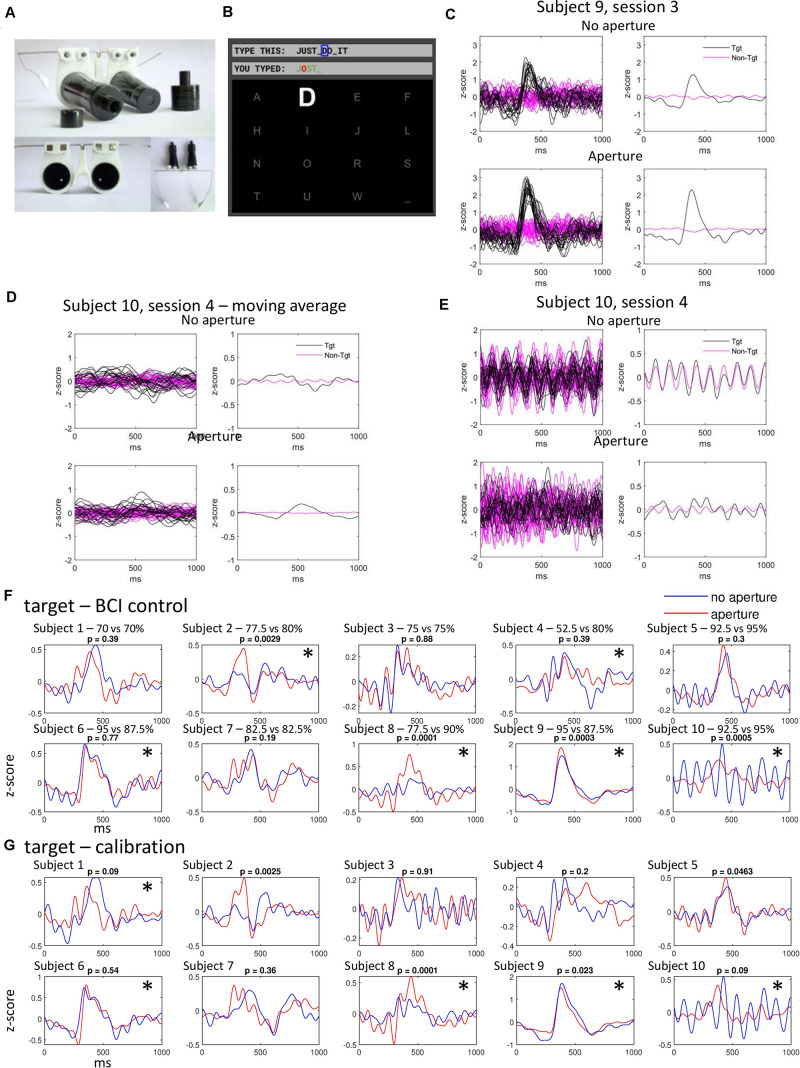
Experimental setup and the ERPs in response to targets and non-targets. **(A)** Aperture headset. **(B)** Computer screen with text characters in a 4 by 4 arrangement. **(C)** ERPs in a subject with target response increased during wearing the aperture. In the panels on the left, each line represents an average ERP for 5 stimuli. The panels on the right show the averages for all responses. Black and magenta lines correspond to target and non-target responses, respectively. Top panels correspond to the no-aperture condition, bottom panels to the aperture condition. The same conventions are used in panels **(D,E)**. **(D)** ERPs in a subject with a strong entrainment of responses to non-targets. A 50-point moving average was used to suppress this response. **(E)** The same data as in **(D)** but without the application of moving average. The 9.1-Hz response to non-targets is prominent in this case. **(F)** Average ERPs for all subjects during BCI control. Red and blue lines represent no-aperture and aperture conditions, respectively. *P*-numbers refer to the comparison of ERP peak values. Asterisks (*) mark the cases where a significant difference was found between the target and non-target ERPs using a randomization test applied to SVM-classifier results. Online classification accuracy (no-aperture vs aperture) is listed, as well. **(G)** Average ERPs for all subjects during calibration. Conventions as in **(F)**.

The pilot experiment was conducted as a part of our ongoing experiments on a P300 BCI of a traditional design. Ten healthy subjects performed a traditional P300 task or switched to wearing a binocular aperture that restricted their sight to the central visual field (Figure 1).

The experimental procedure was approved by the research ethics committee of Samara State Medical University (protocol #204, December 11, 2019). All subjects gave informed consents to their participation in the study. Ten healthy subjects, all males, aged 19 years, right-handed, with right dominant eye determined with Miles test (Miles, 1929), with visual acuity of 1.04 ± 0.18 (mean ± standard deviation) and 1.09 ± 0.18 for the left and right eyes, respectively, measured with Huvitz CCP-3100 projector (HUVITZ Co., Ltd, South Korea) using Cyrillic letters.

The experiments were conducted in a half-dimmed quiet room. Participants sat in front of a 40 × 70 cm computer monitor. The distance from the eyes to the screen was 80 cm. The BCI system included an NVX-36 amplifier, NeoRec software (MKS, Russia), OpenViBE software (Renard et al., 2010), VIBRAINT software (IT Universe Ltd), and an aperture headset (IT Universe Ltd). EEG channels P3, Pz, P4 were recorded according to the international “10–20” system. The left earlobe was used as the reference channel and the right earlobe as the ground. We used a textile cap with gel-based Ag/AgCl wired electrodes MCScap-E (MKS, Russia). Following the requirements of NVX-36 amplifier, each electrode’s impedance was kept below 30 kΩ. The sampling rate was set to 250 Hz. The EEG signal from the NVX-36 amplifier was received by OpenViBE Acquisition Server software and then transmitted to VIBRAINT software for processing. For online spelling, 1,000-ms EEG segments were used that immediately followed stimulus onset. EEG was bandpass filtered with a 4th order Butterworth IIR zero-phase filter in the band 1.0–15.0 Hz. No procedure was applied for removal of eye-blinking or other artifacts. In the experiments with the aperture, the aperture headset was fixed to the head in a way resembling wearing regular glasses (Figure 1A). The aperture tubes were coated with an anti-glare spraying on the inside. The aperture opening was 5 mm in diameter. The distance from the openings to the eyes was 12 cm. The angular size of each character was 2.1 degrees and 4.2 degrees when enlarged. When using the aperture, the angular size of the field of view ranged from 3.2 to 5.7 degrees, which corresponded to the pupil diameter range from 1 to 8 mm. The subjects could see only one character at a time when wearing the aperture.

In our implementation of the P300 speller, 16 characters were displayed on a computer screen in a 4 by 4 arrangement (Figure 1B). The characters flashed randomly, one at a time. When flashing, the character doubled in size (i.e., linear dimensions), and its brightness increased by 80%. Flashes occurred every 110 ms (i.e., 9.1 Hz). Flash duration was 60 ms. A calibration session was run first that lasted approximately 4 min. During the calibration, subjects consecutively looked at 10 characters (“A,” “D,” “E,” “F,” “L,” “S,” space, “W,” “U,” and “O”). Each character flashed 10 times, intermingled with non-target stimuli. Calibration was performed both with and without the aperture, prior to the corresponding BCI sessions.

Following the calibration, subjects used the BCI to generate a ten-character phrase (“JUST DO IT”), either with the traditional P300 speller or while wearing the aperture. Classification was performed with a support vector machine (SVM) classifier trained on the calibration data. The classifier was implemented in VIBRAINT software (LIBLINEAR package). Participants were instructed to look at the target character and avoid making head movements. For every character to be spelled, each screen item flashed 10 times. Next, the classifier predicted the target stimulus based on these data. The classification result was displayed on the screen. For example, in the experimental session shown in Figure 1B, the classifier correctly determined “J,” “S,” “T,” and space, and confused “U” with “O.”

The order of experimental conditions alternated during four daily recording sessions. Subjects 1–9 started with the no-aperture condition in sessions 1 and 3 and started with the aperture condition in sessions 2 and 4. Subject 10 started with the no-aperture condition in sessions 1 and 2 and with the aperture condition in sessions 3 and 4. No clear effect of condition order was found.

## Aperture Effect on ERPs

The comparison of ERPs across the experimental conditions (with and without the aperture) was conducted offline. EEG signals were preprocessed for this analysis by applying a bandpass filtered in the range 1.0–15.0 Hz with a 4th order Butterworth IIR zero-phase filter. For each recording session, EEGs on each channel were standardized to *z*-scores. The aperture and no-aperture recordings were combined for this standardization. (Additionally, we conducted separate standardization for the aperture and no-aperture conditions and obtained similar results). We used Pz channel to assess ERPs in different conditions.

Figures 1C–E shows two examples of recordings for the no-aperture and aperture conditions. In the subject whose data are shown in Figure 1C (subject 9, session 3 of BCI spelling), target ERPs were stronger in the aperture condition than in the no-aperture condition. A 9.1-Hz response evoked by non-targets can be also noticed (top right, magenta trace). Figures 1D,E shows the data from the subject (subject 10, session 4 of BCI spelling) with a particularly strong, clearly periodic at 9.1 Hz response to non-targets (Figure 1E, top). This response could be suppressed by a 50-point moving average (Figure 1D). Following the application of the moving average, high-frequency components were removed from the target response, as well. The slow component of the target ERP was stronger for the aperture condition and was shifted in time relative to the target ERP in the no-aperture condition (Figure 1D, left).

Figure 1F shows average ERPs (all sessions combined) exhibited during BCI spelling in all ten subjects, and Figure 1G shows average ERPs for the calibration sessions. The results are similar for the BCI spelling and calibration sessions. Changes in ERPs can be noticed that resulted from wearing the aperture (compare blue and red curves). To assess these changes, we first determined the time points at which average ERPs peaked for each condition. We next measured the values for these time points for the individual responses (5 consecutive ERPs averaged) and assessed the across-condition difference using Wilcoxon signed rank test. This paired difference test was used because the sequences of characters were the same in the non-aperture and aperture condition. The results were mostly the same when using the unpaired Wilcoxon rank-sum test. With this approach, differences were found in 4 subjects (subjects 2, 8, 9, and 10) for the BCI sessions, and in 4 subjects (subjects 2, 5, 8, and 9) for the calibration sessions. In subjects 2, 8, and 5, ERP peaks increased when wearing the aperture. In subject 9, the peak increased during the BCI sessions and decreased during the calibration sessions. As to subject 10, in this subject, target ERPs were qualitatively different during the no-aperture and aperture sessions: the average ERP was strongly oscillatory without the aperture whereas the oscillatory component was removed by wearing the aperture.

**FIGURE 2:**
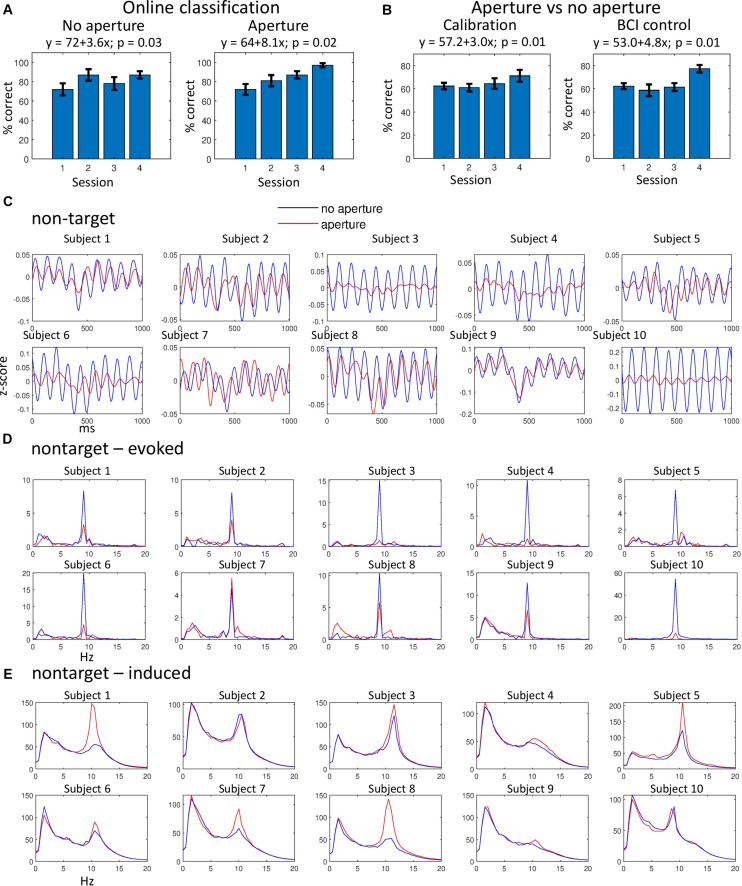
Classification results and analysis of non-target ERPs. **(A)** Online classification results for the aperture (left) and no-aperture (right) conditions. Bars represent mean classification accuracy for all subjects, for four consecutive sessions. Error bars represent standard errors. The results of multiple linear regression are shown that represent accuracy as a function of training day. *P*-values for the regression are given, as well. **(B)** Discrimination of the aperture versus no-aperture ERPs for the calibration (left) and BCI (right panels) sessions. Conventions as in **(A)**. **(C)** Average non-target ERPs for all subjects. **(D)** Evoked ERP spectra for the data in **(C)**. **(E)** Induced ERP spectra for the data in **(C)**. In **(C–E)**, data from BCI sessions were used. Very similar results were obtained for the calibration sessions (not shown). Blue lines correspond to the no-aperture condition and red lines to the aperture condition.

In addition to these changes in ERP peak values, changes in ERP shapes resulting from wearing the aperture are visible in the plots of Figures 1F,G. Yet, these changes are individually variable. Thus, in subjects 1, 2, 5, and 7, the positive response peaked earlier in the aperture condition whereas in subjects 4 and 6 it peaked earlier in the no-aperture condition. To assess the significance in such changes, we compared the no-aperture and aperture ERPs using an SVM. Half of the responses (averages of 5 ERPs) were used to train the SVM and the other half for classification. The classification results are shown in Figure 2B. Statistical significance of this classification was assessed using a randomization test (Fisher, 1935; Edgington and Onghena, 2007) where condition labels (no-aperture and aperture) were randomly shuffled 1,000 times to obtain the statistical samples for comparison of classification results. A subject was deemed as exhibiting a change in ERP shape when wearing the aperture if the SVM detected a statistically significant difference (*p* < 0.05, randomization test) for at least 3 sessions. By this classification, wearing the aperture resulted in a change in ERP shape in 6 subjects (subjects 2, 4, 6, 8, 9, and 10) during the BCI sessions and 5 subjects (subjects 1, 6, 8, 9, and 10) during the calibration sessions. Thus, in around half of the subjects changes in ERPs were observed after putting on the aperture in each type of session (calibration or BCI spelling).

Although the average accuracy of online classification slightly improved from 81.0 ± 2.6 (mean ± standard error) in the no-aperture condition to 84.3 ± 2.9 in the aperture condition, this improvement was not statistically significant for our sample of ten subjects (*p* = 0.35, Wilcoxon signed-rank test). Yet, statistically significant trends were found for the accuracy to improve with training days, more so for the aperture condition (Figure 2A). We also observed a trend for the no-aperture and aperture ERPs to become more different from each other with continued training (Figure 2B).

Our analysis of non-target ERPs revealed a clear entrainment of cortical activity to non-targets presented at 9.1 Hz. The presence of 9.1-Hz periodicity is clear in the average non-target ERPs (Figure 2C). These responses to non-targets diminished during wearing the aperture (compare red and blue curves in Figure 2C) although some periodicity remained, indicating that the flashes of non-targets were not completely abolished by the aperture. A particularly strong decrease in the non-target response was observed in subject 10, whereas in subject 7 no such decrease was observed. For all other subjects ERPS evoked by non-targets were reduced in the aperture condition.

To investigate non-target ERPs further, we computed the spectra of evoked responses (Başar and Bullock, 1992; Tallon-Baudry et al., 1996; David et al., 2006) by calculating fast Fourier transform (FFT) for each ERP (i.e., the same traces as the ones used to calculate average ERPs in Figure 2C), calculating the mean for these spectral representations and then calculating the absolute value (Figure 2D). The evoked-response spectra contained sharp peaks at 9.1 Hz, which were lower in the aperture condition for all subjects except subject 7. Spectral peaks at 9.1 Hz were also present in the target-ERP data, and they decreased in the aperture condition (not shown).

We then conducted an analysis of the spectra of induced responses by calculating FFTs, taking their absolute values and then averaging (Figure 2E). Except for subject 10, these spectra did not contain the 9.1-Hz peaks but instead they contained peaks at ∼10 Hz, which corresponded to alpha activity asynchronous to stimulus periodicity. Curiously, these peaks were higher for the aperture condition than for the no-aperture condition in all subjects except for subjects 2 and 10. The induced-response spectra were virtually the same for the target responses (not shown).

## Discussion

Our pilot experiment showed that subjects could operate a P300 speller while wearing an aperture that restricted their visual field to central vision. With this method, the interference from non-target stimuli was reduced in all subjects except one (subject 7), as evident from average ERPs and evoked-response spectra. Moreover, in half of the subjects (or more, depending on the inclusion criterion), we observed changes in cortical responses to target stimuli. In two subjects (subjects 2 and 8) that was an increase in ERP amplitude. We also observed changes in ERP shapes, such as an earlier positive response in the aperture condition (e.g., subject 1) and a conversion of a mixture of target and non-target responses into a clean response pattern (subject 10).

While these preliminary observations support our hypothesis that removal of non-target stimuli could help better classify targets in the BCI-speller paradigm, we did not observe a statistically significant improvement in the online classification for our experimental settings and for the small (*N* = 10) sample of healthy subjects aged 19. There are several reasons for this result. First, the amplitude of target ERPs did not change substantially enough for classification accuracy to be affected. Second, even though responses to non-targets decreased in the aperture condition, alpha activity increased which was asynchronous to stimulus periodicity but acted as noise that hindered classification. Third, non-target ERPs of clear and consistent shapes could be utilized by the classifier to improve the decoding. All these factors should be considered in future studies that should explore various BCI settings, different age groups, and different neurological conditions.

Thus, our implementation of the reduced visual field idea could be improved in the future. Looking at the screen through a fixed-sized aperture is uncomfortable, and this problem could be addressed using a variable-size aperture that narrows the visual field only when needed. Additionally, the aperture could be applied to one eye only and the subject could close the other eye when focusing on the target. Furthermore, the same effect could be achieved with an algorithm that reduces the flicker of non-targets as ERP data accumulates, and eventually stops it. A hybrid BCI that incorporates eye tracking (Koo et al., 2014; Shishkin et al., 2016; Hong and Khan, 2017) could be also used for this purpose. Indeed, once the fixation point is detected by an eye-tracker, flashes of non-target items could be suppressed. With such a method, the BCI could be controlled without producing head movements, which were necessary in our aperture-based implementation.

Regardless of the technical details of how this approach could be implemented, we see several advantages in a speller where only the central visual field is used for stimulus delivery. The first advantage is that distractors do not interfere with selective spatial attention (Clark and Hillyard, 1996; Hillyard and Anllo-Vento, 1998; Luck et al., 2000; Ordikhani-Seyedlar et al., 2016). This consideration is particularly important when using the speller in patients who may have impaired attention in addition to the other neurological conditions (Vieregge et al., 1999; Phukan et al., 2007; Nazhvani et al., 2013; Yadav et al., 2014). The second advantage is the reduction of visual fatigue, which is a common problem for BCI spellers (Boksem et al., 2005; Käthner et al., 2014). Finally, the third obvious advantage is that, when the number of non-target stimuli is reduced, the speller works faster.

One could argue that, since we are suggesting that non-target stimuli could be eliminated completely, the same could be achieved with an eye tracker and/or head-position tracker: once the gaze angle is reliably detected, spelling could be performed without the need to use a BCI. While this solution could be practical in many cases, recording brain activity is still very useful (Shishkin et al., 2016) because it allows to better assess the subject’s intention, level of attention, and the brain state. Additionally, eye/head tracking is not always possible. We therefore suggest that the most versatile approach would be using as many control signals as possible and selecting the appropriate ones for each concrete task (Hong and Khan, 2017).

Even if the visual field reduction does not happen to be practical in some cases, it could be still useful for the assessment of an individual subject’s ability to control a BCI. For example, in our study, target-stimulus ERPs were unchanged for 6 subjects wearing the aperture, whereas they were affected in 4 other subjects, which suggests that the latter subjects focused better on the target in the absence of distractors. In one of these 4 subjects, responses to non-targets were extraordinarily strong in the regular P300-BCI sessions possibly because of the strong engagement of exogenous attention by the distractors. The removal of distractors dramatically improved the cleanness of the response to the target stimulus, so at least in this subject wearing the aperture had a major positive effect. With more subjects tested in the future and this kind of paradigm extended to patients, we expect that more useful information will be obtained regarding the usefulness of our approach in different individuals. Even the simple aperture test implemented here could serve as useful control that is quickly run to assess the ERPs with and without distracting stimuli. (Such a control does not require changing the basic P300 paradigm, so it is easy to implement).

In addition to bringing improvements to the BCI speller and providing helpful controls, the paradigm proposed here could be useful as a tool in research on brain mechanisms of visual processing and attention. Thus, it is well known that central and peripheral visual fields have different functional properties (Finlay, 1982; Neville and Lawson, 1987; Berencsi et al., 2005), but this topic has not been sufficiently studied with the BCI approach. Given that BCI research contributes to basic science (Nicolelis and Lebedev, 2009), we expect more fundamental insights on the brain mechanisms with the proposed approach.

## Data Availability Statement

The raw data supporting the conclusions of this article will be made available by the authors, without undue reservation.

## Ethics Statement

The studies involving human participants were reviewed and approved by Research Ethics Committee of Samara State Medical University. The patients/participants provided their written informed consent to participate in this study.

## Author Contributions

ML proposed the idea. LK, VB, AO, AK, VP, and ML designed the experiments. LK, VB, and VP conducted experiments. LK, VB, AO, and ML analyzed data. LK, VB, AK, VP, and ML wrote the manuscript. All authors contributed to the article and approved the submitted version.

## Conflict of Interest

VB was employed by the company IT Universe Ltd, Samara, Russia. The remaining authors declare that the research was conducted in the absence of any commercial or financial relationships that could be construed as a potential conflict of interest.
